# Exploring Emotional Regulation in Psychosis: A Qualitative Study of Patient Perspectives

**DOI:** 10.1192/j.eurpsy.2025.2261

**Published:** 2025-08-26

**Authors:** E. Rolland-Carlichi, C. Baeyens, C. Dondé, B. Gouache, C. Bortolon

**Affiliations:** 1 Univ. Grenoble Alpes, Univ. Savoie Mont Blanc, LIP/PC2S; 2 Univ. Grenoble Alpes, GIN; 3 INSERM, U1216, Grenoble; 4 Psychiatry Department, CH Alpes-Isère, Saint-Egrève; 5 Psychiatry Department, CHU Grenoble Alpes; 6 C3R – Centre Référent Réhabilitation Psychosociale et Remédiation Cognitive, CH Alpes-Isère, Grenoble; 7 Institut Universitaire de France (IUF), Paris, France

## Abstract

**Introduction:**

The role of emotional regulation (ER) has emerged as a key mechanism associated with psychosis with important implications for its onset and maintenance. However, knowledge about ER in psychosis has been predominantly quantitative, often neglecting patients’ own perceptions of the difficulties they experience when dealing with their emotions.

**Objectives:**

To address this gap, we have undertaken a qualitative study to investigate ER in individuals with schizophrenia and schizoaffective disorder using semi-structured interviews. Given the qualitative nature of the study, we have no specific hypotheses. Instead, we aim to explore the stages of emotional regulation and the factors that influence ER in people with schizophrenia and schizoaffective disorder, aligning our investigation with existing theoretical models.

**Methods:**

Patient recruitment and data collection are in progress. Thematic analyses will be carried out.

**Results:**

Results will be presented during the European Congress of Psychiatry.

**Conclusions:**

We expect that this study will provide a deeper understanding of ER from the individuals’ perspectives and enrich ER literature and theoretical models related to clinical populations. Ultimately, this research aims to contribute to the development of quantitative studies to test new hypotheses and design individual-tailored psychological interventions aimed at improving ER in patients with schizophrenia and schizoaffective disorder.

**Disclosure of Interest:**

None Declared

